# The complete mitochondrial genome of the *Megopis sinica* white (Coleoptera: Cerambycidae: Prioninae)

**DOI:** 10.1080/23802359.2019.1699461

**Published:** 2019-12-12

**Authors:** Ranran Su, Xiaoyun Wang

**Affiliations:** Guangxi Key Laboratory of Agric-Environment and Agric-Products Safety, National Demonstration Center for Experimental Plant Science Education, College of Agriculture, Guangxi University, Nanning, China

**Keywords:** *Megopis sinica*, Prioninae, mitochondrial genome

## Abstract

The complete mitochondrial genome of the *Megopis sinica* White was sequenced. The 15,689 bp long genome has the standard metazoan complement of 38 genes. These genes contained 13 protein-coding genes, 22 transfer RNA genes, 2 ribosomal RNA genes and 1 control reigion. The nucleotide composition of the *M. sinica* White mitogenome was A: 37.8%, C: 18.6%, G: 11.7%, T: 32.0%. The A + T was 69.8%.

## Introduction

The *Megopis sinica* is a vital pest on fruit and ornamental trees, apple, hawthorn, date, persimmon, chestnut, walnut, pepper and willow etc. (Zhang et al. 2017). The larvae eat food in the cortex and xylem of the branches and the tunnel is irregular and filled with dung debris, which weakens the trees (Lim et al. 2014; Zhang et al. 2017). Mitochondrial genome sequences are essential for a deeper understanding of the evolution of Cerambycidae and identification of larva species (Liu et al. 2018; Wang and Tang 2018; Wang et al. 2019). Here, the complete mitochondrial DNA (mtDNA) genome of *M. sinica* was elucidated which has not been reported before.

In this study, specimens of *M. sinica* were collected from the Qingxiu Mountain (22°47′N, 108°23′E) of Nanning City (Guangxi Province, China). The total genomic DNA was extracted following the modified CTAB DNA extraction protocol and stored at Guangxi Key Laboratory of AgricEnvironment and Agric-Products Safety (The city of Nanning, China) with sample number of SZHT0603G055. Then library was constructed and pair-end was sequenced (2*150 bp) with HiSeq (Illumina, San Diego, CA). Approximately 13.63 G of raw data and 13.59 G of clean data were obtained for sequence assembly by SPAdes (version 3.9) (Bankevich et al. 2012).

The complete mitochondrial genome of *M. sinica* is a closed circular molecule 15,689 bp in length (GenBank accession number MN594765) and constitutive of 38 genes. These genes contain 13 protein-coding genes (PCGs), 22 transfer RNA (tRNA) genes, 2 ribosomal RNA (rRNA) genes, and 1 control region (D-loop). The single non-coding control region (D-Loop) is 863 bp in length. The nucleotide composition of the *M. sinica* mitogenome was A (37.8%), C (18.6%), G (11.7%), T (32.0%). The A + T content was 69.8%, showing strong AT skew.

**Figure 1.**
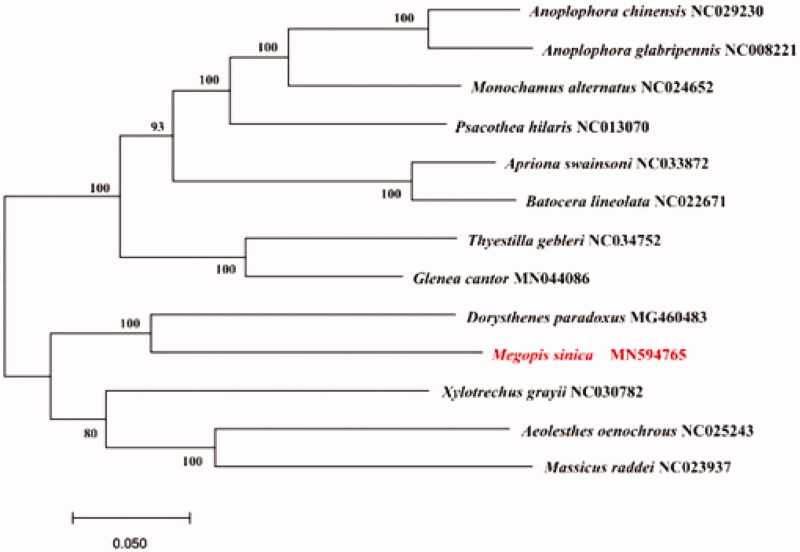
Neighbor-joining phylogenetic tree of *Megopis sinica*, other Prioninae, Cerambycinae and Lamiinae beetles. The complete mitochondrial genome was downloaded from GenBank and the phylogenic tree was constructed by Neighbor-Joining method with 1000 bootstrap replicates. MtDNA accession numbers used for tree construction are as follows: *Megopis sinica* (MN594765), *Anoplophora chinensis* (NC029230), *Anoplophora glabripennis* (NC008221), *Monochamus alternatus* (NC024652), *Psacothea hilaris* (NC013070), *Apriona swainsoni* (NC033872), *Batocera lineolata* (NC022671), *Thyestilla gebleri* (NC034752), *Glenea cantor* (MN044086), *Dorysthenes paradoxus* (MG460483), *Xylotrechus grayii* (NC030782), *Aeolesthes oenochrous* (NC025243), *Massicus raddei* (NC023937).

Molecular Evolutionary Genetics Analysis Version 7.0 (MEGA7.0) was used to make phylogenetic analysis among Prioninae, Cerambycinae and Lamiinae species by Neighbor-Joining method with 1000 bootstrap replicates (Sudhir et al. 2016). The results showed that mtDNA of *M. sinica* had a close relationship with that of *Dorysthenes paradoxus* (Figure 1).
